# Cross-reactivities and cross-neutralization of different envelope glycoproteins E2 antibodies against different genotypes of classical swine fever virus

**DOI:** 10.3389/fvets.2023.1169766

**Published:** 2023-04-27

**Authors:** Wei-Tao Chen, Hsin-Meng Liu, Chia-Yi Chang, Ming-Chung Deng, Yu-Liang Huang, Yen-Chen Chang, Hui-Wen Chang

**Affiliations:** ^1^School of Veterinary Medicine National Taiwan University, Taipei, Taiwan; ^2^School of Veterinary Medicine, Graduate Institute of Molecular and Comparative Pathobiology, National Taiwan University, Taipei, Taiwan; ^3^College of Bioresources and Agriculture, Animal Health Research Institute, Tamsui, Taiwan

**Keywords:** classical swine fever, E2 glycoprotein, cross-reaction, cross-neutralizing activity, genotypes

## Abstract

Classical swine fever (CSF) is a highly contagious swine disease caused by the classical swine fever virus (CSFV), wreaking havoc on global swine production. The virus is divided into three genotypes, each comprising 4–7 sub-genotypes. The major envelope glycoprotein E2 of CSFV plays an essential role in cell attachment, eliciting immune responses, and vaccine development. In this study, to study the cross-reaction and cross-neutralizing activities of antibodies against different genotypes (G) of E2 glycoproteins, ectodomains of G1.1, G2.1, G2.1d, and G3.4 CSFV E2 glycoproteins from a mammalian cell expression system were generated. The cross-reactivities of a panel of immunofluorescence assay-characterized serum derived from pigs with/without a commercial live attenuated G1.1 vaccination against different genotypes of E2 glycoproteins were detected by ELISA. Our result showed that serum against the LPCV cross-reacted with all genotypes of E2 glycoproteins. To evaluate cross-neutralizing activities, hyperimmune serum from different CSFV E2 glycoprotein-immunized mice was also generated. The result showed that mice anti-E2 hyperimmune serum exhibited better neutralizing abilities against homologous CSFV than heterogeneous viruses. In conclusion, the results provide information on the cross-reactivity of antibodies against different genogroups of CSFV E2 glycoproteins and suggest the importance of developing multi-covalent subunit vaccines for the complete protection of CSF.

## 1. Introduction

Classical swine fever (CSF) is a highly contagious World Organization for Animal Health (WOAH) notifiable disease that causes significant economic losses. A devastating CSF has been reported in Central and South America, Europe, Asia, and Africa (1–5). Even though areas can be declared CSF-free, the re-emergence of CSF and emergence of new sub-genotypes of classical swine fever virus (CSFV) have been reported (6). In Japan, outbreaks of G2.1d CSFV in pig farms and wild boars in Gifu City in 2018 were re-emerged after 26 years of CSF-free status (6–8) indicating the difficulty in eradication of the disease.

Clinical signs of CSF are determined by the virulence of the viral strain, age, health condition, and immune responses of pigs and can be divided into peracute, acute, subacute, chronic, and subclinical (3, 9, 10). The common pathological findings in the acute phase are hemorrhage and petechiae in multiple organs with necrotizing tonsillitis and enteritis (11, 12). The most prominent histopathological changes in chronic CSF are lymphoid depletion and lymph node necrosis (13). Subclinical CSF, resembling a persistent infection, is caused by a transplacental transmission during mid-gestation periods (14, 15). Infected piglets can be asymptomatic but persistently shed the virus, becoming a source of virus (11, 16).

Classical swine fever virus, belonging to the family *Flaviviridae* and genus *Pestivirus*, is a single positive-strand RNA virus. CSFV carries a genome of ~12.3 kbp, encoding one continuous open reading frame (ORF) flanked by two non-translated regions (NTR) on both sides. The ORF encodes a polypeptide precursor of approximately 3,898 amino acids (aa) that can be cleaved into 12 mature proteins, including four structural proteins, namely nucleocapsid protein (C), enveloped glycoproteins (E) E^rns^, E1, and E2, and eight non-structural (NS) proteins, namely N-terminal protease (N^pro^), p7, NS2, NS3, NS4A, NS4B, NS5A, and NS5B (17–19). Among the CSFV proteins, the E2 protein is the most immunogenic and essential for inducing neutralizing antibodies and protecting against lethal challenge (20). It has been demonstrated that the removal of certain glycosylation sites of the E2 protein significantly reduced the immunogenicity of the protein and increased its virulence (21, 22). There are four immunogenic domains at the C-terminus of the E2 protein, which can be divided into a less conservative B/C domain (690–779 a.a.) and a conservative A/D domain (780–859 a.a.). Several linear epitopes were identified in these domains (23), such as ^772^LFDGTNP^778^ at the tail of domain B/C (24) and ^829^TAVSPTTLR^837^ recognized by the monoclonal antibody (mAb) WH303 (25). At the N-terminus of the B/C domain, four residues at positions ^709^P, ^713^E, ^725^G, and ^738^I/V have been identified as important for antigen–antibody interactions (26).

Substitutions can cause dramatic topology changes and might abolish antibody binding (27). It has been shown that specific glycosylation or the lack of E2 glycoprotein through point mutation and deglycosylation of the highly virulent Shimen strain at position 986 could result in a lower virulence (22). In this study, deglycosylation of the E2 protein at the ^986^NYA^988^ glycosylation site resulted in a decrease in E2 dimerization, which affected viral interactions with cell surface attachment factors, virion stability, and virus replication (22, 28, 29).

Classical swine fever virus can be divided into three genotypes (G1, G2, and G3). Each genotype comprises four to seven sub-genotypes according to the 5’NTR and E2 sequences (17, 18, 30, 31). Among the different genotypes, the nucleotide sequence identities genetically range from 80 to 86%. In the same genotype, there is 86–91% similarity among various sub-genotypes (18). Only the original reference strain, G1, has been reported in North America. The G2 CSFV emerged in Europe in the 1980s. The G3 CSFV has only been identified in Asia (11, 32). Regarding the historical distribution of sub-genotypes, the G1.1 CSFVs were identified in Argentina, Brazil, Colombia, and Mexico. The G1.3 strains were identified in Honduras and Guatemala. The G1.2 and G1.4 strains were identified in Cuba (32–35). Currently, genotype 2, |originating in Central Europe, is the predominant strain. G2.1 CSFV is a moderately virulent genotype compared with high-virulence G1 strains. The G2.1, 2.2, and 2.3 CSFV strains have been reported in Nepal, China, Japan, Korea, and the Middle East. G3 CSFV has only been reported in Asia, with G3.2 isolated in Korea between 1988 and 1999 (36), G3.3 in Thailand between 1988 and 1996 (1), and G3.4 in Japan and Taiwan (37). In Taiwan, the G3.4 strain was gradually replaced by the G2.1 CSFV. This was suggestively due to the superior replication and infectivity of the G2 virus compared with the G3 CSFV (1). However, the mechanism responsible for genotype switching has not been completely investigated.

Extensive vaccination programs have been used to control CSF in endemic regions, with varying degrees of success. Live attenuated vaccine (LAV) generally performs well against homologous strain CSFV infections. However, conflicting results of various degrees of protection against heterologous strains have been debated (38–40). Even after extensive vaccination with C-strain LAV, frequent CSF outbreaks have been reported in China. The reported strains include G1.1, G2.1, G2.2, G2.3, and the newly emerged sub-genotypes G2.1b, G2.1c, and G2.1d (41–44). The newly emerged clades of subgenotype G2.1 are moderately virulent and more dominant, arguing the efficacy of the C-strain G1-based vaccine.

To study the cross-reaction and cross-neutralizing activities of antibodies against different genotypes of E2 glycoproteins, ectodomains of G1.1, G2.1, G2.1d, and G3.4 CSFV E2 glycoproteins derived from the HEK293 mammalian expression system were generated to mimic the integrity of E2 glycoproteins. These E2 glycoprotein-based in-house ELISAs were developed to evaluate the cross-reactivity of a panel of immunofluorescence assay (IFA)-characterized sera derived from Lapinized Philippines Coronel strain live attenuated vaccine (LPCV) immunized pigs. These ELISA performances were compared with that of a commercialized CSFV ELISA. Hyperimmune mouse serum against these CSFV E2 glycoproteins was generated to detect the neutralizing activity against different genogroups of CSFV.

## 2. Materials and methods

### 2.1. Cells and virus

**Figure 1:**
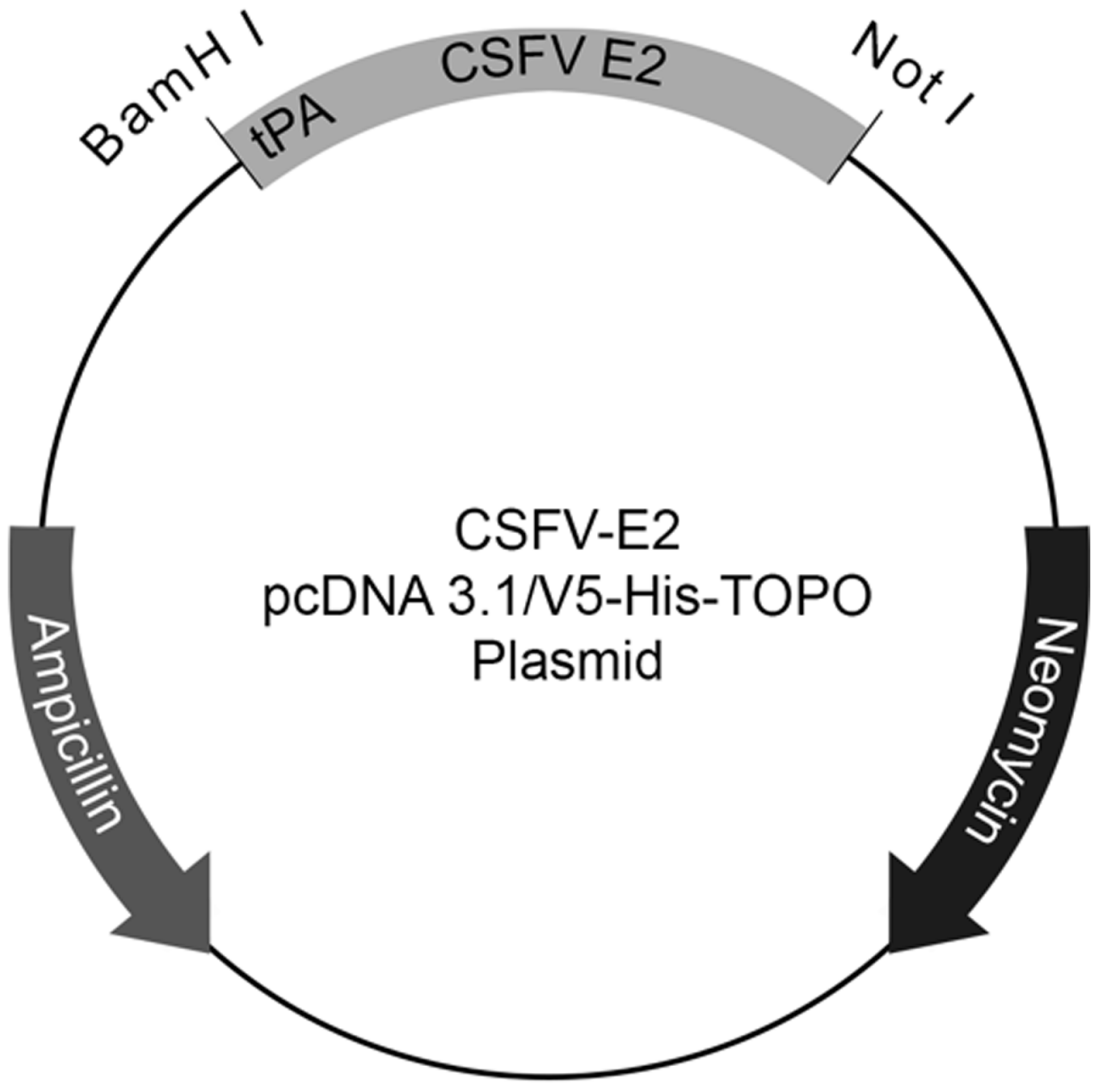
Schematic plasmid map of the recombinant CSFV E2 construct. Sequences of E2 modified by truncation of the transmembrane domains and addition of the human tissue plasminogen activator (tPA) sequence at 5′-end with two restriction enzyme sites, NotI and BamHI, at 3′ and 5′ end of the sequences, respectively, were cloned onto the pcDNATM 3.1/V5-His TOPO^®^ TA mammalian expression vector.

Sequences of the E2 encoded region from G1.1 (GenBank Accession No. AAS20416.1), G2.1a (GenBank Accession No. LC425854.1), G2.1d (GenBank Accession No. AY554397.1), and G3.4 (GenBank Accession No. AY646427.1), modified by truncation of the transmembrane domains and addition of the human tissue plasminogen activator sequence at the 5′-end with two restriction enzyme sites, NotI and BamHI, at the 3′ and 5′ ends, respectively, were synthesized by Genscript Corporation (Piscataway, NJ, United States). The modified sequences were digested and ligated into the pcDNA 3.1/V5-His TOPO TA mammalian expression vector (Invitrogen, Carlsbad, CA, United States) at BamHI and NotI restriction sites (Figure 1).

The plasmids obtained were transfected into HEK 293 cells with PolyJet (SignaGen Laboratories, Frederick, MD, United States) and selected by culturing in DMEM high-glucose culture medium (Gibco, USA) containing 1.5% geneticin (G418) (Gibco) and 10% FBS (Gibco). Once stable cell lines were developed, the cells were placed into 175 T flasks and cultured with Freestyle 293 expression medium (Gibco) for five days for supernatant collection.

### 2.2. Immunocytochemistry staining for E2 detection

The expression of E2 glycoproteins was detected by fixing the cells in a 96-well-plate with 80% acetone (Avantor, PA, United States) for 20 min on ice. After air-drying and washing with 200 μL of PBS, 100 μL of anti-V5 antibody (Invitrogen; 1:1,000 dilution) was added to each well and incubated at room temperature (RT) for 1 h. Each well was washed six times using 200 μL PBS. In each well, 100 μL of Dako REAL EnVision antirabbit/mouse horseradish peroxidase (HRP)-conjugated antibody (Dako, CA, United States; 1:10 dilution) was added and incubated at RT for 1 h. The signals were detected using 3,3′-diaminobenzidine (DAB) (Dako) following the manufacturer’s instructions. Results were evaluated using an inverted light microscope.

### 2.3. Sodium dodecyl sulfate–polyacrylamide gel electrophoresis and western blot

The E2 glycoproteins were mixed with NuPAGE LDS sample buffer (Thermo Fisher Scientific, Waltham, MA, United States). For the denatured samples, NuPAGE Sample Reducing agent (Thermo Fisher Scientific) was added and incubated at 95°C for 10 min. The samples were then separated by SDS-PAGE using a Bio-Rad Mini-PROTEIN electrophoresis system (Bio-Rad, Hercules, CA, United States) with a 10% separating gel and 17% stacking gel, following the manufacturer’s recommendations. The proteins were transferred to a polyvinylidene fluoride (PVDF) membrane (Bio-Rad) and blocked with 5% skim milk (Beckton, Dickson and Company, MD, United States) in 5% tris-buffered saline and polysorbate 20 (Tween 20) (TBS-T) (Genestar, Beijing, China) at RT for 1 h. followed by 1 h. of WH303 (APHA Scientific, United Kingdom; 1:1,000 dilution) or anti-V5 (Novex, Invitrogen; 1:5,000 dilution), and 1 h. of Goat-anti-mouse HRP conjugated secondary antibody (Jackson ImmunoResearch, PA, United States; 1:10,000 dilution) with three washes of TBS-T between each incubation. The results were visualized using Clarity Western ECL Blotting Substrates (Bio-Rad) and a ChemiDoc XSR+ Imaging System (Bio-Rad).

### 2.4. Protein affinity-based purification

The collected expression medium was filtered through a 0.22 μm filter to remove any cell debris. The filtered expression medium was then incubated at 4°C overnight with HisPur cobalt resin (10 mL/1 L) (Thermo Fisher Scientific). The resin was collected in a column and washed with 10 resin-bed volumes of sodium-phosphate-based wash buffer. The proteins were eluted by passing five resin-bed volumes of 300 mM imidazole elution buffer through a column. The eluates were concentrated using Amicon Ultra-15 10 kDa concentration tubes (Millipore, Merck, Ireland). The concentration was determined by measuring the UV absorbance at 280 nm using a Take 3 BioTek microplate (Cytation 7, Agilent, Santa Clara, CA, United States).

### 2.5. Indirect immunofluorescent assay of swine serum antibody

PK-15 cells were seeded in a flat bottom 96-well-plate at 80% confluence and infected with the attenuated LPCV (AHRI) virus at a multiplicity of infection of 10. After 72 h of inoculation, the cells were fixed by adding 100 μL of 10% formaldehyde, incubated at RT for 1 h, and air-dried. One hundred microliters of 10% goat serum (Dako) were used as a blocking buffer and were incubated at RT for 1 h. The sera collected from pigs submitted to Veterinary Medicine Diagnostic Center at School of Veterinary Medicine in National Taiwan University for diagnostic needs with or without LPCV immunization history was diluted 80 folds and incubated at RT for 1 h. After washing with PBS six times, fluorescein isothiocyanate (FITC)-conjugated AffiniPure goat anti-swine IgG antibody (Jackson ImmunoResearch; 1:100 dilution) was applied to the microplates for 1 h at RT. After washing with PBS, the cells were mounted with a mounting medium containing DAPI (Abcam, Cambridge, United Kingdom). Fluorescence was observed using an inverted fluorescence microscope.

### 2.6. Commercial and in-house CSFV enzyme-linked immunosorbent assay

A CSFV antibody ELISA kit (BioChek, Berkshire, UK) was used to detect CSFV antibodies in swine serum, following the manufacturer’s recommendations. For different in-house CSFV E2 ELISA, 100 μL purified E2 proteins diluted to 1 ng/microliter in coating buffer (KPL, SeraCare, Milford, United States) was added onto 96-well-plates, following manufacturer’s instructions, and incubated overnight at 4°C. Following removal of the coating buffer, each well was washed six times with 200 μL of wash buffer (KPL, SeraCare). One hundred microliters of blocking buffer (KPL, SeraCare) were added to each well and incubated at RT for 30 min. Swine or mouse blood serum was diluted 80-fold with PBS before adding to each well. After washing with wash buffer, 100 μL of HRP-conjugated goat anti-swine IgG (Jackson ImmunoResearch; 1:1,000 dilution in blocking buffer) or HRP conjugated goat-anti-mouse secondary antibody (Jackson ImmunoResearch; 1:1,000 dilution in blocking buffer) was added and incubated at RT. After 1 h, the plates were washed six times. Fifty microliter ABTS peroxidase substrate (KPL, SeraCare) was added for 3 min following the manufacturer’s instructions. The reaction was halted by adding a 50 μL stopping buffer (KPL, SeraCare). The results were evaluated by measuring the optical density at 405 nm (OD 405) on an EMax Plus microplate reader (Molecular Device, Crawly, United Kingdom). Cutoff values were determined by adding two standard deviations of all IFA-negative samples to the average of IFA-negative samples. Higher OD values were considered to be positive and vice versa.

### 2.7. Mice immunization

Twelve eight-week-old BALB/c mice were randomly separated into three groups. Each group was administered 50 μg of G1.1, 2.1d, or 3.4 CSFV E2 proteins in 0.2 mL of Montanide Gel 01 (Seppic, France) intraperitoneally and boosted with the same dosage at 14, 28, 42, and 56 days post-immunization (dpi). Hyperimmune mouse serum samples of different E2 levels were collected retro-orbitally and at 70 dpi, per the Institutional Animal Care and Use Committee (IACUC) guidelines. All procedures involving animals were performed following the regulations and with permission of the IACUC protocol (No. A10008) at the Animal Health Research Institute (AHRI, Council of Agriculture, Executive Yuan, Taiwan).

### 2.8. Serum neutralizing assay

The serum neutralizing assay was performed as described in previous studies (45). All serum samples were first inactivated at 56°C. Starting from 1:40 dilution, twofold serial diluted sera were incubated with equal amount of100 TCID_50_ of different CSFV genotypes, including LPC/AHRI strain (G1.1) (46), TD/96/TWN strain (G2.1a) (47), and 94.4/IL/94/TWN (G3.4) (32) at 37°C for 1 h, and subsequently added into PK-15 seeded 96-well microplates. At 72 h post-infection, the cells were fixed with 10% formalin for CSFV antigen detection by IFA staining, as previously described (45). The neutralizing titer in the log_2_ of the Ab dilution factor was recorded.

### 2.9. Translational alignment and statistical analysis

Translational alignment of all four E2 sequences were carried out using Geneious 9 (Version 9.1.8).^1^ Data were analyzed using software GraphPad Prism (version 8.4.0) (GraphPad Software Inc., San Diego, CA, United States) and differences were considered significant by *p*-value (*p < 0.05; **p < 0.01).

## 3. Results

### 3.1. Expression and detection of different CSFV E2 glycoproteins

**Figure 2:**
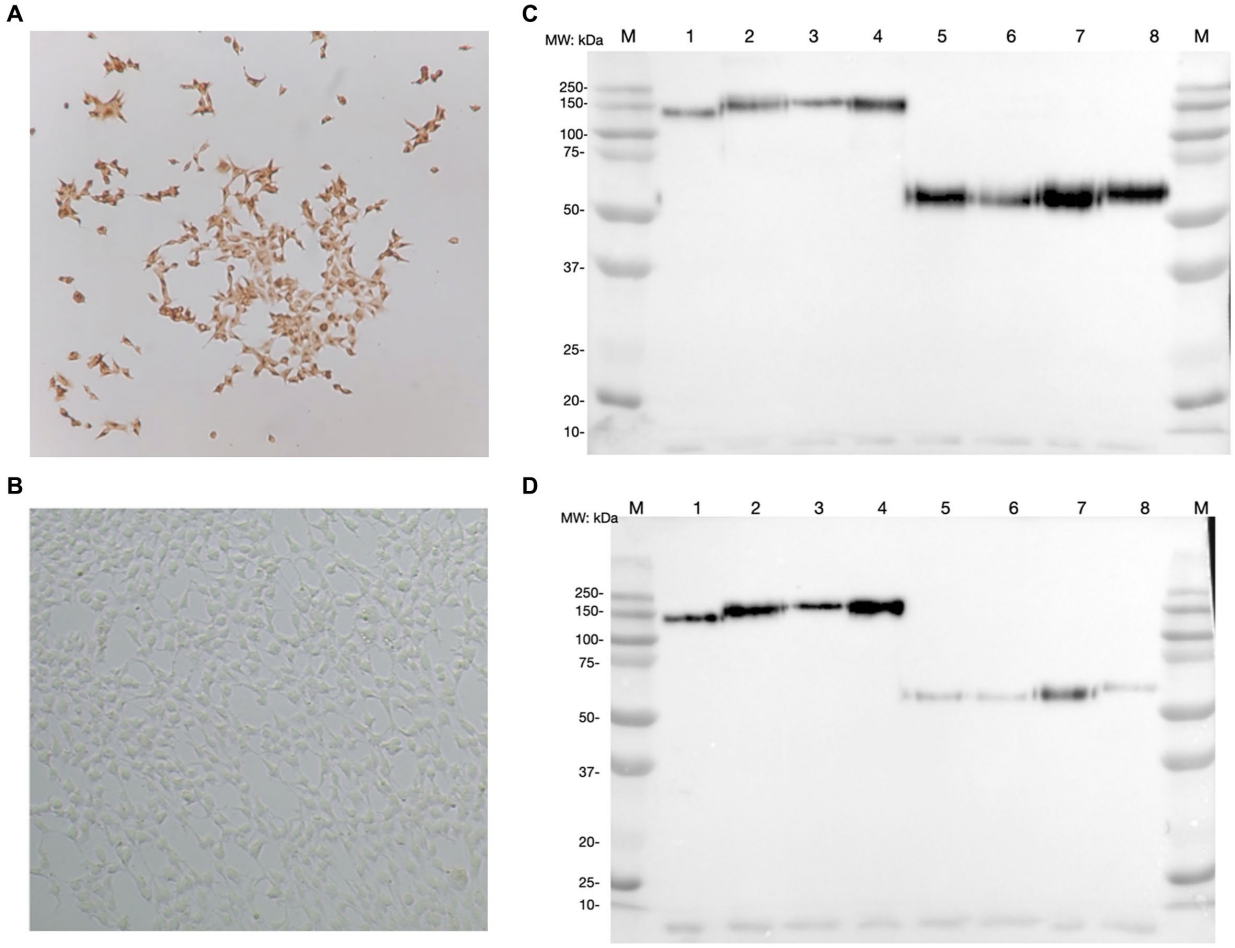
Detection of classical swine fever virus (CSFV) E2 expression by immunocytochemistry staining (ICC) and western blot (WB). **(A)** The result of ICC in detecting the expression of CSFV E2 glycoprotein using anti-V5 antibody. **(B)** The ICC result of mock transfected HEK 293 cells **(C)** Western blot result of different CSFV genotypes of E2 glycoproteins detected by anti-V5 antibody. **(D)** Western blot result of different CSFV genotypes of E2 glycoproteins detected by WH303 antibody. The ICC were performed 7 days after the transfection in HEK 293 cells fixed with 80% acetone for the immunostaining. Proteins after purification with HisPurTM cobalt resin and separated by SDS-PAGE in both naïve and reduced gels were analyzed by WB detected with anti-V5 or anti-WH303 antibodies. The positions of markers are as indicated. 1, CSFV G 1.1 E2 naïve form; 2, CSFV G 2.1a E2 naïve form; 3, CSFV G 2.1d E2 naïve form; 4, CSFV G 3.4 E2 naïve form; 5, CSFV G 1.1 E2 reduced form; 6, CSFV G 2.1a E2 reduced form; 7, CSFV G 2.1d E2 reduced form; 8, CSFV G 3.4 E2 reduced form; M, Marker.

After G418 selection, the expression of each CSFV E2 glycoprotein was successfully detected in HEK293 cells using an anti-V5 antibody. In each CSFV E2 plasmid-transfected cell line, more than 90% of cells were stained positive by ICC (Figures 2A,B). The expression medium collected contained 3–4.5 mg of E2 glycoprotein/L after purification. After protein purification of the supernatant of these CSFV E2 glycoprotein-expressing stable cell lines, proteins migrated to 100 kDa under non-reduction conditions and were suspected as homodimers. Proteins migrated to 50 kDa under reduction conditions corresponding to the predicted size of E2 monomer were confirmed by using an anti-V5 antibody (Figure 2C) and the anti-CSF E2 specific antibodies, WH303 (Figure 2D).

### 3.2. Cross-reactivity of LPCV-induced antibody responses in pigs against different genotypes of CSFV E2 proteins

To investigate the cross-reactivity of the LPC-induced porcine IgG against different genotypes of CSFV E2 proteins, a panel of 177 porcine serum samples from farms with and without an LPC-vaccination history was used. The binding activity of porcine sera against CSFV was first evaluated using IFA on LPC virus-infected PK-15 cells. Under fluorescent microscopic examination, a total of 78 serum samples were positive for IgG against LPC-infected PK-15 cells. Ninety-nine sera were negative.

**Figure 3:**
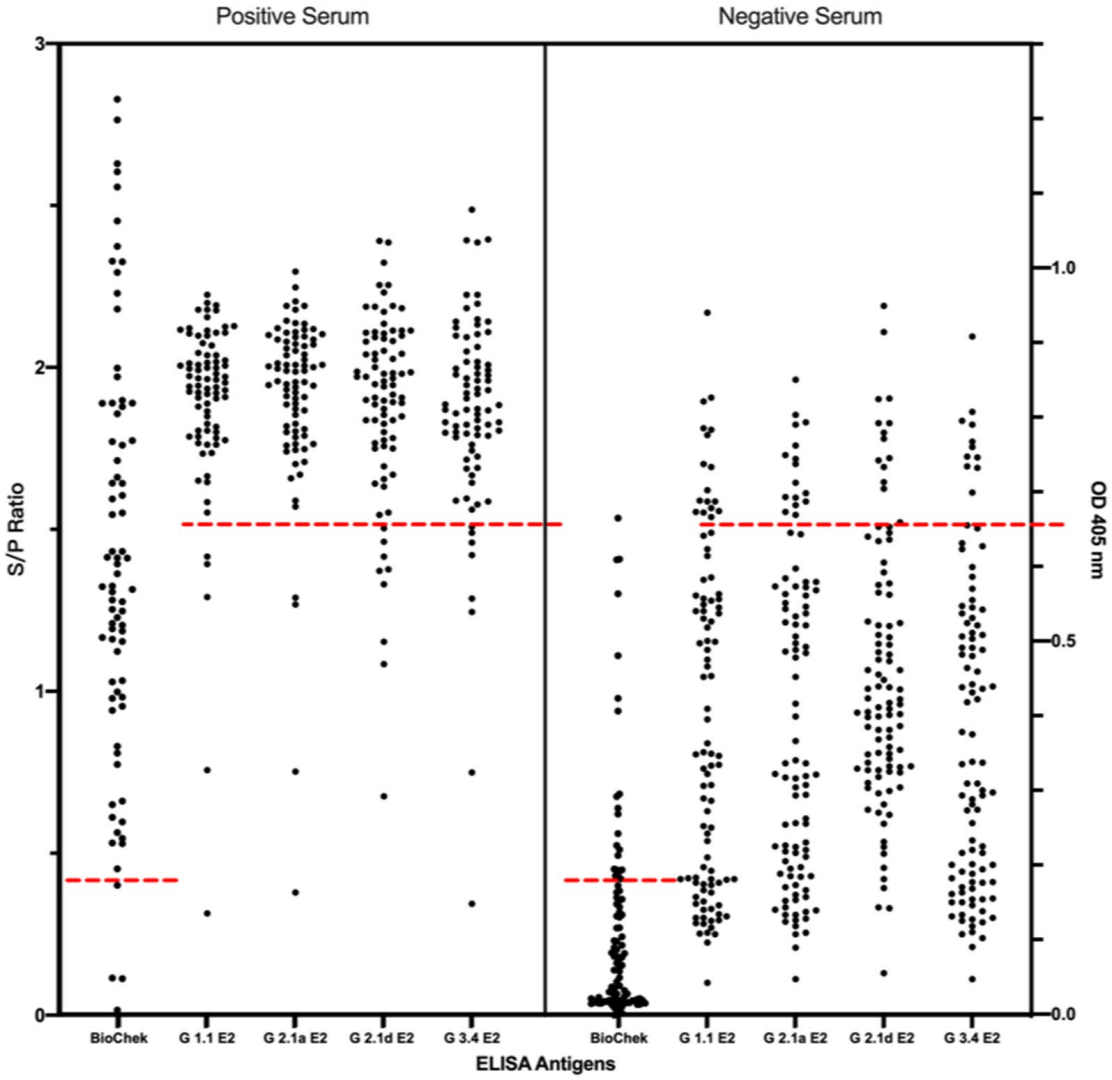
Comparison of cross-reactivities of serums derived from pigs with/without LPC vaccine-immunization among the commercial CSFV E2 ELISA and different four different in-house E2 glycoproteins-based ELISA. Results of the commercial CSFV E2 ELISA and in-house E2-based ELISAs are represented as in S/P ratio and OD 405, respectively. Black round dots on the right column are IFA-confirmed negative serums and dots on the left column are the IFA-confirmed serums. Cut-off values are calculated according to the manufacture’s guideline or the mean value of the negative samples plus two standard deviations (mean + 2SD) for in-house ELISA presented as red dotted lines.

Using the IFA-characterized porcine sera, the cross-reactivity of these porcine sera against different genotypes of CSFV E2 proteins was investigated by ELISAs (Figure 3). The S/P ratio of the commercially available ELISA was calculated following the manufacturer’s recommendations. Using the mean value of the negative samples plus two standard deviations (mean + 2SD) as the cut-off values for in-house CSFV G1.1 E2-based ELISA had a cut-off O.D. of 0.71; the in-house CSFV G2.1a E2-based ELISA had a cut-off value of 0.71; the in-house CSFV 2.1d-based E2 glycoprotein ELISA had a cut-off value of 0.70; and the in-house CSFV 3.4 E2-based glycoprotein ELISA had a cut-off value of 0.66. After evaluating four in-house E2 glycoprotein-based ELISA of the 177 serum samples, all glycoproteins showed comparable sensitivity and specificity to the commercially available CSFV E2 ELISA (Table 1).

**Table 1:** Summarized results of in-house E2 glycoproteins-based ELISAs and the commercial ELISA in detection of IFA-confirmed positive (+) and negative (−) serums against the classical swine fever virus (CSFV) Lapinized Philippines Coronel (LPC) strain in PK-15 cells.

	ELISA result	BioChek	CSFV 1.1 E2	CSFV 2.1 E2	CSFV 2.1d E2	CSFV 3.4 E2
IFA (+) (*N* = 78)	Positive	73	70	72	67	70
	Negative	5	8	6	11	8
IFA (−) (*N* = 99)	Positive	14	8	9	13	11
	Negative	85	91	90	86	88
Sensitivity		0.936	0.828	0.860	0.828	0.860
Specificity		0.859	0.919	0.909	0.869	0.889
Accuracy		0.893	0.875	0.885	0.849	0.875

### 3.3. Virus cross-neutralizing test for different CSFV E2 immunized mice

**Figure 4:**
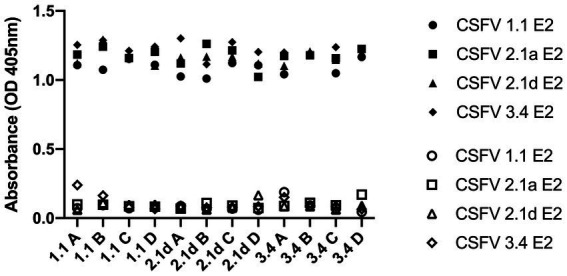
Cross-reactivity of IgG before and after G 1.1, 2.1d, and 3.4 E2 immunization in mice against different E2 glycoproteins detected by ELISA. Sera sample were collected retro-orbitally before immunization and at 70 days post immunization with different E2 glycoproteins and anti-E2 antibody levels were measured by ELISA coated with G 1.1, G 2.1a, G 2.1d, and G 3.4 E2 and read at OD 405 nm. The hollow icons represent serum samples collected prior to immunization and solid icons are at 70 days post immunization. Circular shape is against G 1.1 in-house E2-based ELISA, square shape is against G 2.1a in-house E2-based ELISA, triangular shape is against G 2.1d in-house E2-based ELISA, and diamond shape is against G 3.4 in-house E2-based ELISA.

**Figure 5:**
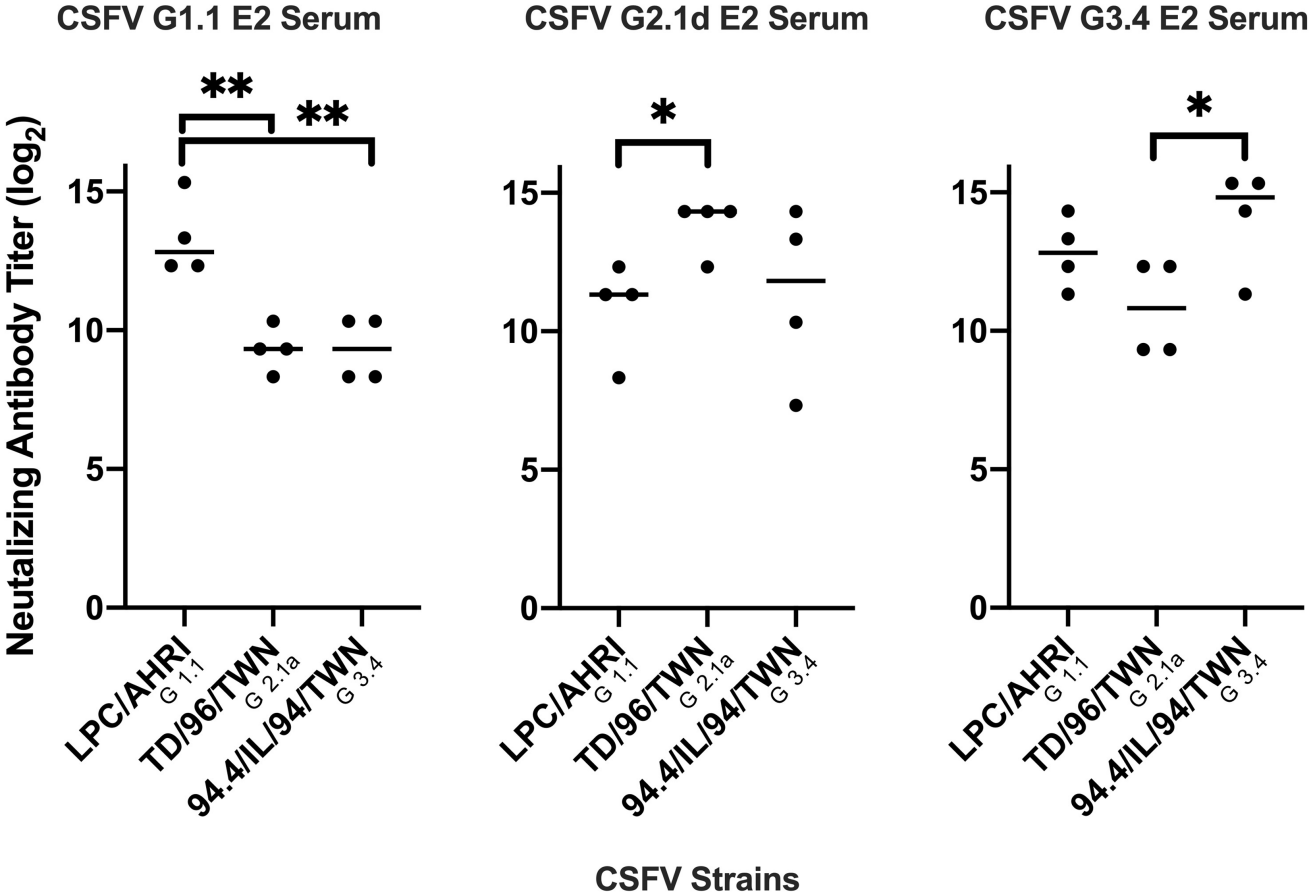
Comparison of different mice serum neutralizing antibody titer against different CSFV genotypes. Mice sera at 70 days after immunization with G 1.1, G 2.1d, or G 3.4 glycoproteins were collected, inactivated at 56°C, incubated with 100 TCID50 of LPC/AHRI strain (G 1.1), TD/96/TWN strain (G 2.1a), or 94.4/IL/94/TWN strain (G 3.4) of CSFV for 1 h at 37°C, and infected PK-15 cells. The highest dilution that is able to stop 50 percent of the cell from infection were recorded. *p < 0.05; **p < 0.01.

After immunization, elevated anti-CSFV E2 IgG levels in the sera of different CSFV E2 immunized mice were detected. There were no differences in the IgG-binding abilities of these sera against homologous and heterologous CSFV E2 proteins (Figure 4). Antibody neutralizing (NA) in the sera of CSFV E2-immunized mice generally exhibited a better NA against the homologous genotype virus than against the heterologous viruses (Figure 5). Mice immunized with CSFV G1.1 E2 glycoprotein presented significantly higher NA titers (average 1:10,240 dilution) against LPC/AHRI strain (G 1.1) infection than the TD/96/TWN strain (G 2.1a) (average dilution 1:640) and 94.4/IL/94/TWN strain (G 3.4) (average dilution 1:640) in sera. In contrast, CSFV G2.1d E2 protein-immunized sera also exhibited significantly higher NA against the homologous TD/96/TWN strain challenge (G2.1a; average dilution 1:14,480) than LPC/AHRI strain (CSFV G1.1; average dilution 1:1810) and higher NA against the 94.4/IL/94/TWN strain (G 3.4) (average dilution 1:2,560). CSFV G3.4 E2 protein-immunized sera exhibited higher NA titer against 94.4/IL/94/TWN strain (G 3.4) (average dilution 1:17,220) than the LPC/AHRI strain (G 1.1) (average dilution 1:7,240) and significantly higher NA against the TD/96/TWN strain (G 2.1a) (average dilution 1:1,810). No detectable NA was detected in any of the sera collected before protein immunization.

### 3.4. The deduced amino acid alignments of different E2 sequences

**FigURE 6:**
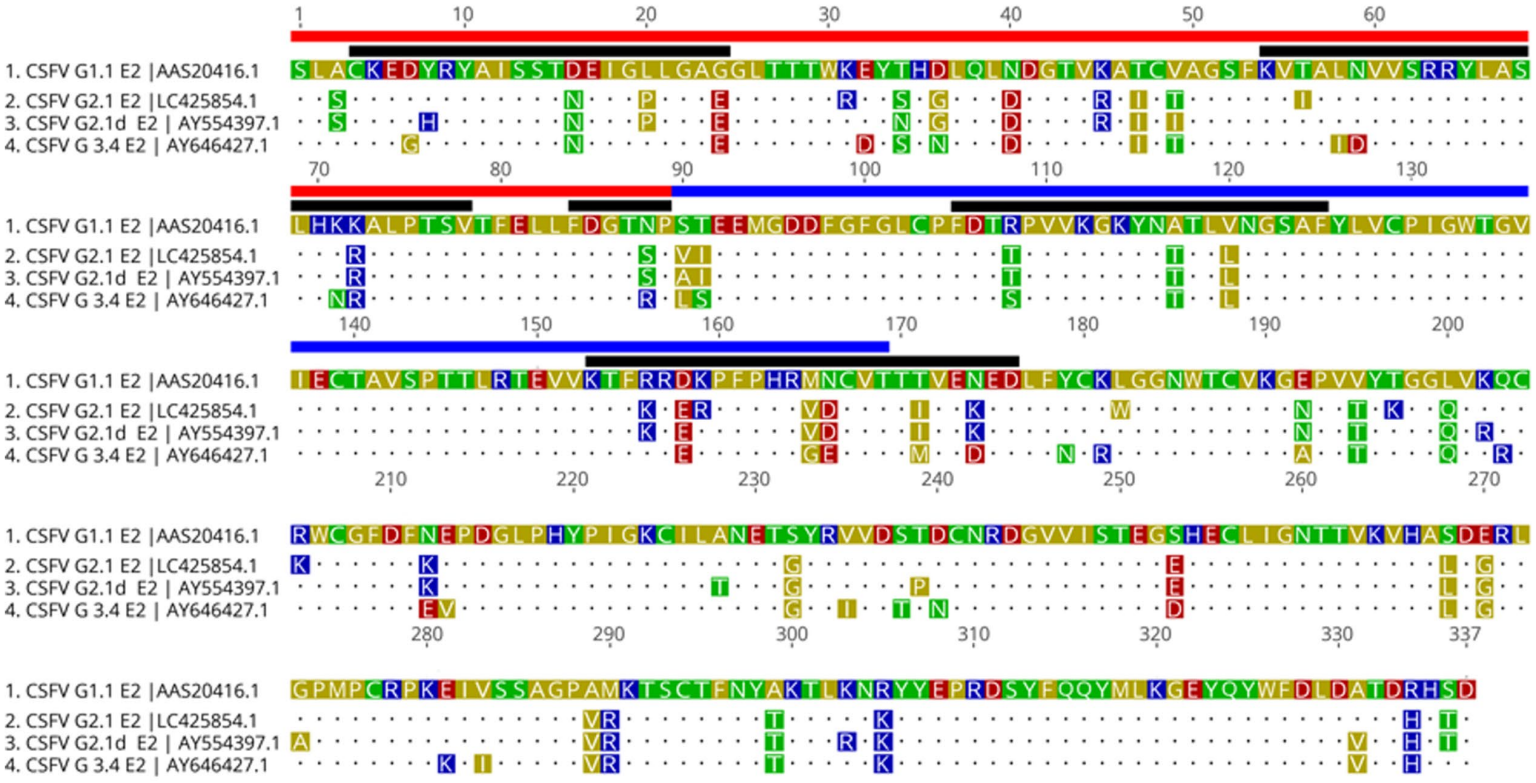
Translational alignment of different genotypes of CSFV E2 sequences. The G 1.1 sequence is used as the reference sequence. The B/C domain are underlined in red and A/D domain in blue. Known epitope region are indicated by black line. The extra predicted glycosylation site of G 2.1a, G 2.1d, and G3.4 are marked by the red box.

The alignment result revealed several variations in known epitope regions of E2 glycoproteins (Figure 6). As compared to the G 1.1 E2, the G 2.1a E2 had substitutions of L709P, G713E, D725G, V738T, T745I, K761R, and N777S in domain B/C, R786T, A795T, V789L, R848K, D850E, K851R, M857V, N858D, T863I, and N866K in domain A/D; the G 2.1d E2 had substitutions of Y697H, L709P, G713E, D725G, V738I, K761R, and N777S in domain B/C,R786T, A795T, V789L, R848K, D850E, K851R, M857V, N858D, T863I, and N866K in domain A/D. The G 3.4 E2 had substitutions of G713E, K720R, D725N, V738T, K760N, K761R, N777R in domain B/C, R786S, A795T, V789L, D850E, M857G, N858E, T863M, and N866D in domain A/D. Also, substitution of A988T present in G 2.1a, G 2.1d and G 3.4 result in an extra glycosylation site compared to G 1.1 of LPC strain (Figure 6; Supplementary Table 1).

## 4. Discussion

Historically, CSF has been controlled by extensive vaccination and complete stamp-out programs. However, outbreaks of G2.1d CSF occurred in a large number of C-strain-vaccinated pig farms in China, highlighting the importance of investigating the cross-reaction and cross-neutralizing activity of immune responses against different genotypes of CSFVs (23, 43). In this study, ectodomains of G1.1, G2.1a, G2.1d, and G3.4 CSFV E2 glycoproteins were successfully generated in a mammalian expression system. Antibodies derived from the G1.1 LPCV-immunized animals were demonstrated to recognize all genotypes of E2 glycoproteins. We also demonstrated that different E2 antibodies exhibited better neutralizing abilities against homologous CSFV than heterogeneous viruses. The results provide information on the cross-reactivity of antibodies against different genogroups of CSFV E2 glycoproteins and suggest the importance of developing multivalent E2 subunit vaccines for CSF protection.

In this study, the high cross-reactivity of LPC-induced porcine IgG against different genotypes of CSFV E2 proteins was confirmed by ELISA. Our results also indicated that the HEK293 cell-derived E2 glycoprotein-based ELISA developed, exhibited comparable sensitivity and specificity to the commercially available CSFV E2 ELISA. Several CSFV E2- and E^rns^-based ELISAs have been developed to evaluate CSFV exposure and immunity in animals with E2 being the most widely adopted and commercially successful ELISA (48–50). Similar to our results, the indirect ELISA based on the Shimen strain (G1.1) E2 expressed by lentivirus-infected Chinese hamster ovary (CHO) cells has been reported to have 92.9% agreement with the viral neutralizing test and 92.2% agreement with the IDEXX blocking ELISA (48). In *Spodoptera frugiperda* (SF21) cells expression system, it has been demonstrated that the Brescia (G1.2) strain, Paderborn (G2.1) strain, and Kanagawa (G3.4) strain E2-based ELISA derived had high Ab binding activities against homologous strain-immunized swine hyperimmune serum (51). However, the E2 protein-based ELISA derived from the *E. coli* expression system had a relative sensitivity of 90.2% and a relative specificity of 55.3% compared with the IDEXX blocking ELISA kit with an overall concordance rate of 80.3% (52). The low specificity of the *E. coli*-expressed E2 protein-based ELISA argues the accuracy of the method.

Regarding cross-protectivity of CSFV vaccines, it has been shown that the C-strain vaccine and the LPC G1-based vaccines could prevent the circulation of most G1 CSFVs in the world and reduce the incidences of G3.2 CSFV in Korea, G3.3 CSFV in Thailand, and G3.4 CSFV in Japan and Taiwan (36, 37, 53). The tissue-adapted version of the C-stain vaccine Riemser vaccine has also been demonstrated to provide complete protection against G2.1 and G3.3, and G2.1c CSFVs (8, 38). However, we have demonstrated herein that hyperimmune serum from CSFV E2 glycoprotein-immunized mice exhibited better neutralizing abilities against homologous CSFV than heterogeneous viruses. Notably, sera derived from mice immunized with the LPC strain E2 (G1.1) had a lower neutralizing antibody titer against G2.1 and G3.4 CSFVs. This is consistent with the previous findings (23, 45). Our results might also explain, at least partially, occasional cases of the new G2.1b and G2.1d sub-genotypes of CSFV infection in a large number of C-strain-vaccinated pig farms in China (54, 55) and the findings of C-strain-based vaccination could provide clinical but not pathological and virological protection against the G2.1d CSFV emerging in China (39). According to our results, we speculated that the antibody induced by the monovalent G1.1 E2 subunit vaccine might not be able to completely neutralize heterogeneous viruses. Since the above-mentioned vaccines are LAVs, further animal experiments to evaluate the cross-protectivity of different genotype E2 proteins against different genotype CSFVs to understand the immune efficacy and protectivity of E2 subunit vaccines are needed.

To investigate potential mutations responsible for the reduction of neutralizing abilities of E2 antibodies against heterogeneous CSFVs, amino acid sequence alignment was performed. Several amino acid substitutions, D705N, L709P, G713E, N723S, and S779A, in the G2.1a and G2.1d E2 sequences reported as antigenic domains responsible for a decrease in the neutralizing ability of heterologous strains (23, 26), were noted. Importantly, these mutations were demonstrated to lead to conformational changes in the antigenic epitope domain covering ^773^FDGTNP^778^ of the E2 protein predicted by SWISS-MODEL as compared with the CSFV G1.1 E2 protein in the present study (Supplementary Figure 1). This domain is a conserved linear B-cell epitope composed of three essential residues ^773^F, ^775^G, and ^778^P, with ^774^D and ^777^N contributing to most of the epitope activity. Replacing of these residues has been demonstrated to abolish or remarkably reduce the reactivity of the epitope (56). We propose that the substitutions and structural alteration of the epitope domains of the E2 protein might be responsible for the differences in the lower neutralizing ability of the G1.1 LPC strain virus against the G2.1a and G2.1d CSFVs. Further investigations, such as mutagenesis assays of E2 proteins, to map critical mutations responsible for the viral neutralization are also needed.

Live attenuated vaccines have been widely used to control CSFV. Among the currently used vaccines to combat CSFV, LAVs are the most common, with worldwide adaptation to region-specific strains or genotypes. However, LAVs have several disadvantages, including acceptance of maternal-derived Ab titer interference (57), lack of DIVA ability, the requirement for low-temperature transportation, and the possibility of virulence reversion (58). Combined with the progress in molecular biology and insight into the pathogenesis of CSFV infections, methods to distinguish vaccinated and clinically infected pigs can be developed using subunit E2 DIVA vaccines. However, variable results of vaccination-challenge experiments and transmission studies on E2 subunit vaccines (59, 60) suggest limited capacity of monovalent CSFV E2 subunit vaccines to provide sterilizing immunity against heterogeneous field CSFV-strains in pigs (59–61). For CSFV subunit vaccine development, multivalent subunit vaccines should be essential based on the reduction in neutralizing ability against heterogeneous CSFV observed in the present study. Using the mammalian expression system of HEK-293 could provide unique opportunities for E2 proteins to process complex multi-dimensional folding and post-translational modifications. Four CSFV E2 proteins covering G1-G3 CSFV with proper mammalian glycosylation and able to elicit neutralizing antibodies against G1–G3 CSFVs generated in this study could be multi-covalent CSFV subunit vaccine candidates.

## Data availability statement

The original contributions presented in the study are included in the article/Supplementary material, further inquiries can be directed to the corresponding author

## Ethics statement

The animal study was reviewed and approved by IACUC protocol (No. A10008) at the Animal Health Research Institute.

## Author contributions

W-TC and H-ML contributed to the data acquisition, analysis, and interpretation. W-TC drafted and revised the manuscript. C-YC, M-CD, Y-LH, Y-CC, and H-WC contributed to the conception, design, and data acquisition. H-WC contributed to the conception and design, revised the manuscript critically for important intellectual content, and approved the final version to be published. All authors agreed to be accountable for all aspects of this work, ensuring that questions related to the accuracy and integrity of any part of this work were appropriately investigated and resolved.

## Funding

This work was supported by the Ministry of Science and Technology, Taiwan, R.O.C. (MOST 109-2313-B-002-016-MY3) and the National Taiwan University (Grant no. 112L894802).

## Conflict of interest

The authors declare that the research was conducted in the absence of any commercial or financial relationships that could be construed as a potential conflict of interest.

## Publisher’s note

All claims expressed in this article are solely those of the authors and do not necessarily represent those of their affiliated organizations, or those of the publisher, the editors and the reviewers. Any product that may be evaluated in this article, or claim that may be made by its manufacturer, is not guaranteed or endorsed by the publisher.

## Footnotes

^1^ http://www.geneious.com
