# Supplementary material for: Cross-reactivities and cross-neutralization of different envelope glycoproteins E2 antibodies against different genotypes of classical swine fever virus
Source: Front Vet Sci. 2023 Apr 27;10:1169766. doi: 10.3389/fvets.2023.1169766 (PMC10172653; doi:10.3389/fvets.2023.1169766)
Supplement: Supplementary file 1 [file Data_Sheet_1.docx]

Supplementary Material

Characterization of Cross-Reactivity of Antibodies against Different Genotypes of Classical Swine Fever Virus Envelope E2 Glycoproteins

Wei-Tao Chen, Hsin-Meng Liu Chia-Yi Chang, Ming-Chung Deng, Yu-Liang Huang, Yen-Chen Chang, Hui-Wen Chang^*^

*** Correspondence:** Hui-Wen Chang: huiwenchang@ntu.edu.tw

# Supplementary Data

## Variants of deduced amino acid of different genotypes (G) CSFV E2 sequences

Translational alignment of all four E2 sequences were carried out using Geneious 9.

## Alignment of different E2 sequence analysis and SWISS-MODEL protein homology modelling of different E2 proteins

Translational alignment of all four E2 sequences were carried out using Geneious 9. Models of respective protein sequences were built using SWISS-MODEL using the crystal structure of the ectodomain of bovine viral diarrhea virus 1 E2 glycoprotein as the template [1,2].

# Supplementary Table and Figure

|  | **G1.1** | **G2.1** | **G2.1d** | **G3.4** |
| --- | --- | --- | --- | --- |
| **3** | **A** | **S** | **S** | **-** |
| **6** | **D** | **-** | **-** | **G** |
| **7** | **Y** | **-** | **H** | **-** |
| **16** | **D** | **N** | **N** | **N** |
| **20** | **L** | **P** | **P** | **-** |
| **24** | **G** | **E** | **E** | **E** |
| **31** | **K** | **-** | **-** | **R** |
| **31** | **E** | **-** | **-** | **D** |
| **34** | **T** | **S** | **N** | **S** |
| **36** | **D** | **G** | **G** | **N** |
| **40** | **N** | **D** | **D** | **D** |
| **45** | **K** | **R** | **R** | **-** |
| **47** | **T** | **I** | **I** | **I** |
| **49** | **V** | **T** | **I** | **T** |
| **56** | **T** | **I** | **-** | **-** |
| **58** | **L** | **-** | **-** | **N** |
| **59** | **N** | **-** | **-** | **D** |
| **71** | **K** | **-** | **-** | **N** |
| **72** | **K** | **R** | **R** | **R** |
| **88** | **N** | **S** | **S** | **R** |
| **90** | **S** | **V** | **A** | **L** |
| **91** | **T** | **I** | **I** | **S** |
| **108** | **R** | **T** | **T** | **S** |
| **117** | **A** | **T** | **T** | **T** |
| **120** | **V** | **L** | **L** | **L** |
| **156** | **R** | **K** | **K** | **-** |
| **158** | **D** | **E** | **E** | **E** |
| **159** | **K** | **R** | **-** | **-** |
| **165** | **M** | **V** | **V** | **G** |
| **166** | **N** | **D** | **D** | **E** |
| **171** | **T** | **I** | **I** | **M** |
| **174** | **N** | **K** | **K** | **D** |
| **179** | **Y** | **-** | **-** | **N** |
| **181** | **K** | **-** | **-** | **R** |
| **182** | **L** | **W** | **-** | **-** |
| **192** | **E** | **N** | **N** | **A** |
| **195** | **V** | **T** | **T** | **T** |
| **197** | **T** | **K** | **-** | **-** |
| **200** | **L** | **Q** | **Q** | **Q** |
| **202** | **K** | **-** | **R** | **-** |
| **203** | **Q** | **-** | **-** | **R** |
| **205** | **R** | **K** | **-** | **-** |
| **212** | **N** | **K** | **K** | **E** |
| **213** | **E** | **-** | **-** | **V** |
| **228** | **A** | **-** | **T** | **-** |
| **232** | **S** | **G** | **G** | **G** |
| **235** | **V** | **-** | **-** | **I** |
| **238** | **S** | **-** | **-** | **T** |
| **239** | **T** | **-** | **P** | **-** |
| **240** | **D** | **-** | **-** | **N** |
| **253** | **S** | **E** | **E** | **D** |
| **268** | **S** | **L** | **L** | **L** |
| **270** | **E** | **G** | **G** | **G** |
| **273** | **G** | **-** | **A** | **-** |
| **281** | **E** | **-** | **-** | **K** |
| **283** | **V** | **-** | **-** | **I** |
| **289** | **A** | **V** | **V** | **V** |
| **290** | **M** | **R** | **R** | **R** |
| **299** | **A** | **T** | **T** | **T** |
| **303** | **K** | **-** | **R** | **-** |
| **305** | **R** | **K** | **K** | **K** |
| **331** | **A** | **-** | **V** | **V** |
| **334** | **R** | **H** | **H** | **H** |
| **336** | **S** | **T** | **T** | **-** |

**Supplementary Table 1.** Variation of deduced amino acid of different genotypes (G) CSFV E2 sequences after translational alignment.


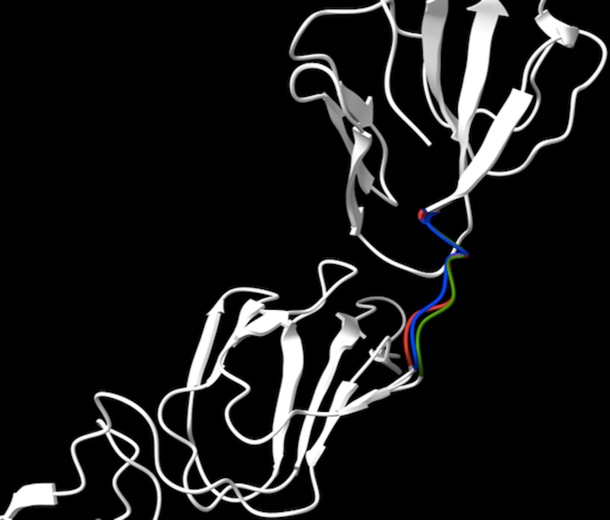


**Supplementary Figure 1.** Translational alignment of different genotypes of CSFV E2 sequences. The SWISS-MODEL was obtained based on the crystal structure of the ectodomain of bovine viral diarrhea virus 1 E2 glycoprotein as the template. The G 1.1 sequence is used as the reference sequence. The B/C domain are underlined in red and A/D domain in blue. Known epitope region are indicated by black line. The extra predicted glycosylation site of G 2.1a, G 2.1d and G3.4 are marked by the red box.

# References

1. Li, Y., Wang, J., Kanai, R., & Modis, Y. Crystal structure of glycoprotein E2 from bovine viral diarrhea virus. Proceedings of the National Academy of Sciences, 2013; 110(17), 6805-6810.
2. Waterhouse A, Bertoni M, Bienert S, Studer G, Tauriello G, Gumienny R, et al. SWISS-MODEL: homology modelling of protein structures and complexes. Nucleic acids research. 2018;46(W1): W296-W303.
